# Run-and-tumble particles learning chemotaxis

**DOI:** 10.1039/d5sm01040c

**Published:** 2025-11-06

**Authors:** Nicholas Tovazzi, Gorka Muñoz-Gil, Michele Caraglio

**Affiliations:** a Dipartimento di Fisica, Dipartimento di Fisica, via Sommarive 14 38123 Trento Italy; b Institut für Theoretische Physik, Universität Innsbruck Technikerstraße 21A A-6020 Innsbruck Austria michele.caraglio@uibk.ac.at

## Abstract

Through evolution, bacteria have developed the ability to perform chemotactic motion in order to find nourishment. By adopting a machine learning approach, we aim to understand how this behavior arises. We consider run-and-tumble agents able to tune the instantaneous probability of switching between the run and the tumble phase. When such agents are navigating in an environment characterized by a concentration field pointing towards a circular target, we investigate how a chemotactic strategy may be learned starting from unbiased run-and-tumble dynamics. Target detection is allowed only during the tumble phase, which qualifies our agents as truly intermittent searchers. We compare the learning performances of agents that sense only the instantaneous concentration with those of two types of agents both having a short-term memory that allows them to perform temporal comparisons. While all types of learning agents develop successful target-search policies, we demonstrate that those achieved by agents endowed with temporal comparison abilities are significantly more efficient, particularly when the initial distance from the target is large. Finally, we also show that when an additional length scale is imposed, for example by fixing the initial distance to the target, the learning agents can leverage this information to further improve their efficiency in locating the target.

## Abstract 2

Run-and-tumble agents able to tune the probability of switching between the run and the tumble phase can learn chemotactic motion when navigating in an environment characterized by a concentration field pointing towards a circular target.

## Graphical abstract

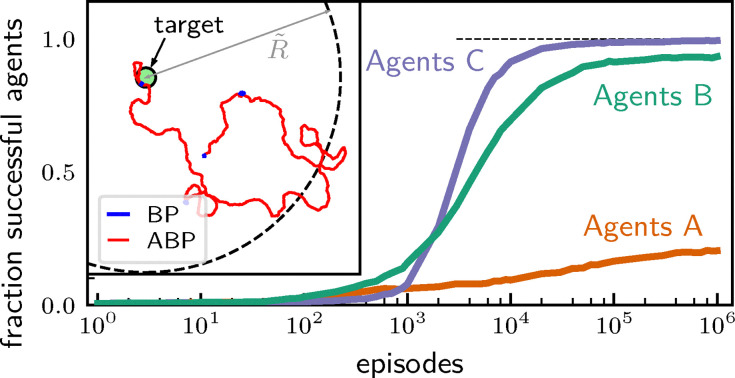

## 1 Introduction

Chemotaxis is a widespread phenomenon in nature. Paradigmatic examples are bacteria foraging nourishment or escaping toxic substrates,^1,2^ phagocytes of the immune system responding to injury or infection,^3,4^ and sperm cells navigating towards the egg.^5^

Several bacteria, including *Escherichia coli*, exhibit movement patterns characterized by an alternating sequence of near-to-straight ‘runs’ at almost constant speed and reorientation events called ‘tumbles’.^2^ The durations of such phases are random variables: for example, *E. coli* in a uniform dilute aqueous medium, display exponentially distributed phase durations, with a mean of 1 and 0.1 seconds for the run and the tumble phase, respectively.^2,6^ However, in the presence of nutrients or other environmental stimuli, chemotactic motion emerges, consisting of a biased random walk with prolonged runs in the preferred direction.^2,6–8^ Bacteria like *E. coli* reach this goal thanks to a complex chemotaxis network that allows them to sense gradients of chemicals by making temporal comparisons on a short time scale.^6,9,10^ Such a network is highly refined and evolutionary optimized, being able to discriminate concentrations in about five orders of magnitude starting from about 3 ligands per cell volume.^6^

As a paradigmatic model of nonequilibrium dynamics, run-and-tumble motion has been extensively investigated in statistical physics, see ref. 11 and references therein. At the single-particle level, the typical theoretical modeling involves a diffusion-drift equation for the one-particle probability density which allows finding exact analytical results.^12–15^ Chemotactic behavior can be included by asserting that the drift velocity in that equation is proportional to the gradient of the scalar field modelling the concentration of chemoattractants,^12^ with a coefficient of proportionality which can be related to the microscopic parameters of the run-and-tumble particle.^16^ The mechanism leading to such behavior is a continuous modulation of the instantaneous tumbling probability as a function of a differential weighting of past measurements of chemoattractant concentration.^12,16^ Within this picture, the steady-state behavior and the optimal chemotactic strategy, *i.e.* the optimal modulation of the tumble rate in response to concentration changes, can be analytically investigated in various regimes.^12,17–19^ It turns out that the optimal strategy depends on properties of the environment and of the individual bacterium, and is therefore highly adaptive.^17^ Other interesting approaches to model bacterial chemotaxis involves active particles whose motion is controlled by an internal clock with thicking depending on the external concentration field.^20^

In this work, to better understand how evolution shaped the search strategies of bacteria, we adopt an approach based on machine learning. In the recent past, reinforcement learning (RL)^21^ and genetic algorithms^22^ have already emerged as powerful tools in active matter research.^23,24^ Focusing on improving the navigation and target-search performances of microswimmers, promising results have been obtained in several situations: smart active agents have been successfully trained to increase their odds of finding stochastically distributed targets of unknown position in homogeneous environments.^25,26^ The potential of RL methods to enable active particles to exploit environmental stimuli in order to bias their motility patterns has been further demonstrated in both steady and turbulent flows,^27–29^ as well as in complex motility fields.^30^ The problem of finding the optimal navigation path between two locations for self-propelled agents which can freely steer has been addressed both in sufficiently complex energy landscapes^31,32^ and in turbulent flows.^33,34^ Robust locomotion strategies at low Reynolds numbers can be learned by simple microswimmers able to modify their shape^35,36^ and more complex flagellated microrobot taking inspiration from sperm cells can learn chemotactic motion by gait switching exploiting deep RL.^37^ Finally, RL algorithms have been even incorporated into an experimental setup providing real-time control of self-thermophoretic active particles in an aqueous solution to steer them towards a given location.^38^

Turning to bacterial chemotaxis, supervised machine learning has recently been exploited to determine optimal memory kernels that filter the information obtained from noisy measurements of ligand concentrations along the trajectory of a run-and-tumble particle, in order to asses increases in concentration signals and consequently trigger tumbles.^39^ The boundary between chemotaxis driven by temporal estimation, typically performed by bacteria, and chemotaxis driven by spatial estimation of gradients, advantageus at larger scales, have been investigated employing both deep RL^40^ and information theory.^41^ Pramanik *et al.* investigated the chemotactic performances of one-dimensional run-and-tumble agents moving in environments with inhomogeneous attractant concentrations, finding that properly tuning the exploration-exploitation trade-off is crucial in certain circumstances.^42^ Interestingly, in a viscous environment involving chemical gradients, biased run-and-tumble dynamics emerges when applying a genetic algorithm to a simple three-bead swimmer able to perform one-dimensional locomotion by adapting the length of the arms connecting the central bead to the external ones.^36^

Here, we consider two-dimensional run-and-tumble agents modelled as intermittent active Brownian particles^26,43^ and initially performing unbiased random dynamics. Exploiting the projective simulation (PS) algorithm,^44^ we show how an efficient chemotactic target-search behavior can be achieved when the agents are immersed in an environment characterized by a single circular target releasing chemicals uniformly into the surrounding space. To better understand the role of temporal comparison, we benchmark the performance of agents that sense only the instantaneous concentration against those equipped with short-term memory, enabling them to perform temporal comparisons.

The target considered in this work is small with respect to the typical length that our agent covers during a run phase. Target detection by bacteria relies on temporal integration of concentration changes over a characteristic timescale of tens to hundreds of milliseconds.^6,9,10^ Thus, a transient increase in attractant concentration when passing over a small target may not last long enough, potentially limiting the bacterium's ability to detect a small target during the run phase. Following the idea that fast motion may degrade perception abilities, we framed our model within the class of intermittent-searcher strategies,^45–48^ which involve agents combining a phase of slow displacement that enables target detection, and a phase of faster motion during which the target cannot be detected but that allows the agent to reach new unvisited regions. In such strategies, the mean search time can be minimized under broad conditions. In particular, it has been shown that there is an optimal duration of the fast nonreactive phase which depends only on the dimensionality of the system and is independent of the details of the slow reactive.^49^

## 2 Model

A circular target of diameter *σ* is placed at the origin of an infinite two-dimensional domain in which the agent is moving, and emits a cue that will be specified later. With two-dimensional run-and-tumble motion in mind, we model our searching agent as a particle that alternates between two distinct phases encoded in the binary variable *ϕ*: a passive phase (*ϕ* = 0), during which the motion of the particle is governed by standard translational diffusion with diffusion coefficient *D*, and an active phase (*ϕ* = 1), during which the particle is also able to self-propel with constant velocity *v* in a given direction described by an angle *ϑ*. The latter also undergoes a diffusion process with rotational diffusion coefficient *D*_*ϑ*_. These two navigation modes are also referred to as the Brownian particle (BP) phase and the active Brownian particle (ABP) phase, respectively. When the particle changes phase, a complete randomization of the self-propultion direction occurs (tumble event). We impose that target detection is enabled only when the agent is in the BP phase. In contrast, during the ABP phase, the agent cannot acquire the target but on the other hand, exploiting its self-propulsion, it can rapidly relocate to a different region in space. This choice makes our agents intermittent searchers.^45–48^ However, here we note that, beyond this modeling choice, our work differs from the usual target search problems considered in the broad literature of intermitten-search, which typically addresses scenarios involving multiple, randomly or regularly distributed targets that do not emit detectable cues.

We endow our run-and-tumble particles with some perception abilities and investigate implications on their target-search behavior. In particular, we consider three sets of agents (A, B, and C) with different sensorial skills. Agents of type A are able to sense the distance from the target, *r*, which is a proxy for the concentration of chemicals released by nourishment. At each time step *t*, their state *s*^A^_*t*_, is characterized by the tuple *s*^A^_*t*_ = (*ϕ*_*t*_,*r*_*t*_), where *ϕ*_*t*_ represents the current phase and *r*_*t*_ represents the distance from the target. Agents of type B cannot exploit the absolute value of the target distance but they have the ability to sense concentration differences on a short time scale, which is attained by evaluating if, after an integration time step Δ*t*, the agent reaches a position that is closer to the target. Their state *s*^B^_*t*_, is given by the tuple *s*^B^_*t*_ = (*ϕ*_*t*_,*ω*_*t*_), with *ω*_*t*_ being a binary variable that equals one if the agent moved closer to the target (*r*_*t*_ < *r*_*t*−Δ*t*_) and zero otherwise. Since the distance *r* from the target center is not included in the state representation, agents of type B do not have an instantaneous perception of having reached the target. However, as we will show in the Results section, this does not compromise their ability to develop an effective policy, as the information encoded in the variable *ω* is sufficient for them to determine when to switch to the passive phase in order to detect the target. Agent of type B are thus mimicking the perception abilities of the bacteria that, like the *E. coli*, are able to make temporal comparison of concentrations. Finally, agents of type C combines the sensorial skills of both types A and B agents, their state *s*^C^_*t*_ being given by the tuple *s*^C^_*t*_ = (*ϕ*_*t*_,*r*_*t*_,*ω*_*t*_). Following the RL framework,^21^ given its current state *s*_*t*_, the agent responds with an action *a*_*t*_, receiving a reward if this action proves beneficial. Here, the action involves deciding whether to retain the current phase or switch to the other one, thus also randomizing the self-propulsion direction. This decision follows a probabilistic rule, with *p*_*t*_ the probability of switching phases. Note that *p*_*t*_ is not fixed: it depends on the agent's current state, *s*_*t*_. The entire set of these state-dependent probabilities constitutes the agent's policy, which is updated during learning to maximize the total reward (see Methods section for details). We note here that the switch from tumble to run is often considered an event that is independent of the environment. However, there is experimental evidence suggesting that bacteria can modulate the amount of reorientation during tumbles by controlling their duration.^50,51^ Thus, independence of tumbling from the environment should not be taken for granted, and despite the limitations of a simplified model, a learning scheme such as the one we propose could provide a method to assess whether an environment-independent passive-to-active rate is indeed the optimal strategy.

Integrating the above concepts into the standard ABP model^52^ within a homogeneous environment yields the following Langevin equations, discretized according to the Itô rule:1
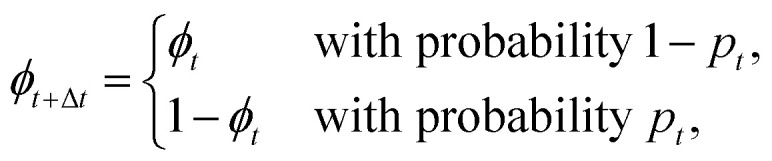
2
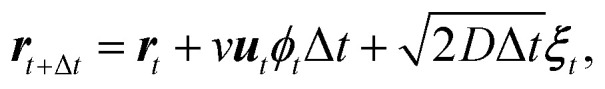
3
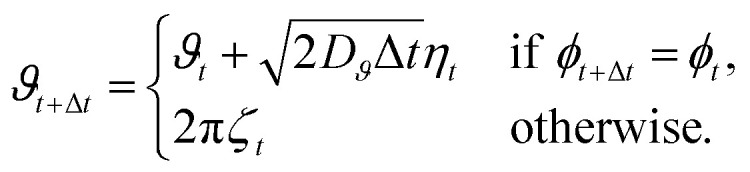


Here, ***r***_*t*_ = (*x*_*t*_,*y*_*t*_) is the position at time *t* and ***u***_*t*_ = (cos *ϑ*_*t*_,sin *ϑ*_*t*_). Additionally, the components of the vector noise ***ξ***_*t*_ = (*ξ*_*x*,*t*_,*ξ*_*y*,*t*_) and of the scalar noise *η*_*t*_ are independent Gaussian random variables with zero mean and unit variance. Finally, *ζ*_*t*_ is a random variable distributed according to a uniform distribution in the interval [0,1]. Note that when the particle is in the passive Brownian phase (*ϕ*_*t*_ = 0), spatial evolution is decoupled from the orientational diffusion of the self-propulsion. In the following, we set the unit of length as the target size *σ* and the unit of time as *τ* := *σ*^2^/4*D*, the typical time a passive particle requires to traverse this distance. The model has then two dimensionless parameters: the Péclet number Pe := *vτ*/*σ*, representing the importance of active relocation with respect to passive diffusion during an ABP phase, and the persistence ℓ* := *v*/*D*_*ϑ*_*σ*, reflecting the persistence of directed motion in the ABP phase.

The learning process is split into several episodes, each episode starting with the agent placed at a certain position ***r***_ini_, uniformly sampled in the annular region defined by *σ*/2 < *r*_ini_ ≤ *R̃*, and having a certain self-propulsion direction *ϑ*_ini_, uniformly sampled in the interval (0,2π]. Here, *R̃* = 10*σ* represents the maximum initial distance from the target that has been considered. Introducing a finite cut-off *R̃* for the maximum initial agent-target distance is necessary to design a well-defined learning protocol: In the limit *R̃* → ∞, there would be no finite episode duration that allows a finite fraction of agents to obtain a reward by finding the target, which would make learning ultimately impossible. We also use *R̃* to define the maximum distance beyond which agents of type A and C can no longer detect the distance to the target. Beyond this distance, in the case of type A and C agents, for each *ϕ* (and *ω*), all the values of *r* are collected in a single state. Note, however, that type B and C agents perform temporal comparison also for *r* > *R̃*, so that the variable *ω*_*t*_ is always well defined. Each episode ends either when the agent meets the target (*r* ≤ *σ*/2) or, in any case, after a time *τ̃* = *τ*, which, on average, is long enough to allow our run-and-tumble particles to travel a distance larger than *R̃* provided that Pe ≳ 10 and that active and passive phases have a typical duration smaller than *τ̃*. Each time the agent finds the target it earns a positive reward. Since rewards are given only when the target is found, the agent learns to minimize the search time and, in effect, to optimize search efficiency.

**Fig. 1:**
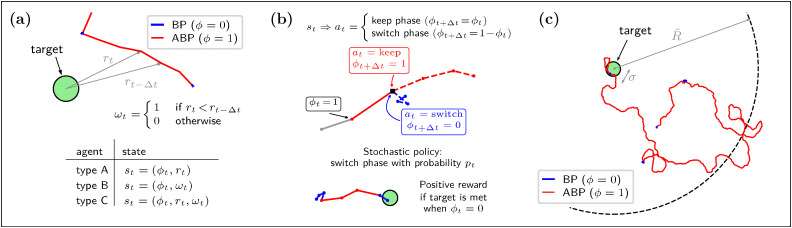
Schematic illustration of the model. (a) Definition of the state for the three types of agents. The agent behaves as a Brownian particle (BP) when the phase is *ϕ* = 0 and as an active Brownian particle (ABP) when *ϕ* = 1; (b) at each integration step, the agent may switch phase with a probability *p*_*t*_ which depends on the state *s*_*t*_. Target acquisition is only possible during the BP phase (*ϕ* = 0); upon successful acquisition, the agent receives a positive reward. (c) Example of a typical trajectory ending with target acquisition, obtained with the model parameters used throughout this work and an agent following the the initial policy.

Fig. 1 presents a schematic illustration of the model together with a typical trajectory, generated with the parameters used in this work, that ends in target acquisition for an agent following the initial policy.

Finally, we note that in intermittent-search theory, periodic boundary conditions are typically assumed.^48^ This corresponds to the case of regularly spaced targets in an infinite domain. In contrast, we opted to consider a single target in an infinite domain to keep the signal perceived by the agent as simple as possible, avoiding situations in which signals from multiple sources need to be processed by the agent. Furthermore, to maintain simplicity, we also avoided introducing hard walls that confine the searching domain. Interactions of active particles with hard boundaries involve several additional phenomena, such as accumulation at walls or trapping by wedges,^52^ and their proper modeling would require accounting for hydrodynamic interactions. Although these aspects are interesting, considering them goes beyond the goals of the present study.

## 3 Results

In the following, the reported results are evaluated by averaging the different quantities over a sample of *N* = 10^4^ independent learning agents with Pe = 100 and ℓ* = 1. The latter parameters are such that, similar to real bacteria, our agents are alternating phases of standard diffusion and runs during which the direction of the motion slowly varies. The details of the learning algorithm PS are reported in the Methods section. Here, we just note that the initial policy is chosen so that the ratio of time spent in the active phase to that in the passive phase matches the ratio of time *E. coli* bacteria spend in the run phase *versus* the tumble phase. Namely, at each time step Δ*t* = 10^−4^*τ*, the initial policy assigns probabilities of phase switching as 10^−2^ and 10^−3^ for passive and active phases, respectively. Consequently, the typical displacement due to diffusion during passive phases is 0.1*σ*, while the typical displacement due to active propulsion during active phases is about 10*σ*. Thus, in the initial unbiased dynamics, active propulsion outweighs passive diffusion by about two orders of magnitude, indicating that our model, with appropriately chosen parameters, provides a reasonable description of run-and-tumble bacterial motion in a homogeneous environment and a suitable starting point to learn a behavioral policy in presence of signals released by a target. For an illustration of a typical trajectory followed by an agent behaving according to the initial policy, see Fig. 1c.

**Fig. 2:**
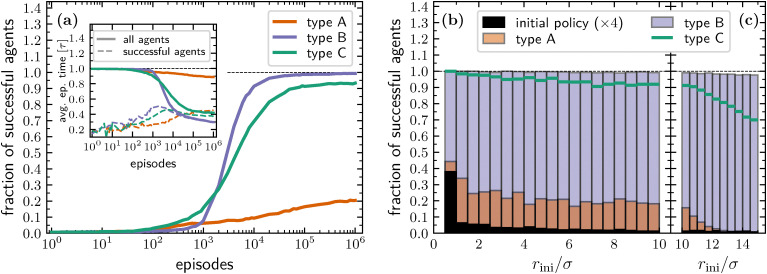
(a) Fraction of successful agents as a function of the number of episodes for both type A (orange line), type B (blue line), and type C (green line) agents. Inset: Average episode time as a function of the number of episodes both for all agents (full lines) for successful agents only (dashed lines). (b) Fraction of successful agents as a function of initial distance from the target, *r*_ini_, evaluated considering the initial policy (black bars) and the ones learned at episode 10^6^ for type A (orange bars), type B (blue bars), and type C (green lines) agents. To improve visibility, values related to the initial policy are multiplied by a factor 4. (c) Same as panel (b) but with agents having an initial distance to the target uniformly sampled in the annular region defined by *R̃* < *r*_ini_ ≤ 3*R̃*/2. This agents do not contribute to the learning process but they are used to test how the learned policy works when the initial distance is larger than *R̃*.

We start by considering how the fraction of agents ending an episode with target acquisition evolves during the learning process. For all types of agents we observe a steady increase in the performances, with agents able to sense their phase and the distance to the target (type A) showing an improvement from about 0.6%, corresponding to the adopted initial policy, to about 20% at the 10^6^-th episode, see Fig. 2a. Thanks to their different perceptor, making the agents aware if over a time step Δ*t* they are getting closer or not to the target, agents of type B display an impressive learned efficiency with about 99% of found targets at the 10^6^-th episode. Type C agents exhibit performances close to those of type B, reaching about 94% of found targets at the 10^6^-th episode. The performances of type B and C agents are even more remarkable if one considers that, in the active phase with Pe = 100, over a single time step Δ*t* the distance covered due to the self-propulsion mechanism is equal to the average distance covered by translational diffusion. Thus, being the latter in a completely random direction, the exploitable information transmitted to the additional perceptor in the form of the binary variable *ω* is greatly reduced. Interestingly, the learning rate of type B agents is initially slower when compared to that of agents that include the absolute distance to the target in their state. However, after approximately 10^3^ episodes, they begin to rapidly improve their performance, outperforming the other types of agents within the following 10^3^ episodes and reaching an efficiency close to 1 already after 10^5^ episodes. This behavior is likely due to the state representation of the agents: while knowing the distance to the target allows for policy improvement already in the first episodes, the larger number of states that type A and C agents can visit slows down learning over the long term. However, since the state representation of type C agents is an extension of that of type B agents, we expect that type C agents will also reach an efficiency similar to that of type B agents at later episodes. On the other hand, we cannot predict the maximum efficiency that type A agents could achieve. Unfortunately, due to the exponential slowing down of the learning efficiency, it becomes computationally impractical to check these observations by extending the number of episodes by another one or two orders of magnitude.

Typically, the efficiency of a search process is characterized by the mean first-passage time.^53^ In the current problem this observable cannot be calculated analytically. However, since our episodes terminate either upon target acquisition or after reaching the full duration of the episode *τ*, we can define the typical first-passage time as the average episode time, including those episodes that terminate without target acquisition. As the number of successful episodes increases, the typical first-passage time decreases, reaching values at episode 10^6^ that are about 10%, 80%, and 60% smaller than their initial ones for type A, B, and C agents, respectively. On the other hand, at the beginning of the learning process, the average time to reach the target conditioned to successful episodes exhibits relatively large fluctuations due to the limited number of agents that are able to reach the target when the policy is still unoptimized (as is clear from the main panel of Fig. 2a). Counterintuitively, this observable is initially increasing, and, only for type B and C agents, in between about 10^2^ and 10^3^ episodes it starts to decrease, see inset of Fig. 2a. This result is due to the fact that at the beginning of the learning process, finding the target is mainly a matter of favorable initial conditions, *i.e.* a value of *r*_ini_ slightly larger than the target radius *σ*/2, see Fig. 2b. On the other hand, while learning, more and more agents having a larger initial distance from the target become successful (Fig. 2b), which initially results in an increase of the average time to reach the target. However, at large episode numbers, type B and C agents become more and more able to exploit the active phase in order to quickly cover the distance separating them from the target, with the consequent decrease in the average time to reach the target. Interestingly, while for particles of type A the final fraction of successful events shows a clear peak for small initial distances and a rather flat distribution for other distances, the same observable computed for type B and C agents remains close to one and only very slowly decreases with increasing distance, see Fig. 2b.

An interesting question is how the learned policy performs when the initial distance exceeds *R̃*. To reply this question, Fig. 2c also reports the fraction of successful agents starting the search at an initial distance from the target larger than the cut-off *R̃* used during the learning process. In this case, for all types of agents, the number of agents that reach the target within a time *τ* decreases as the initial distance increases, partly because the initial distance grows while the duration of the search episode remains fixed. This effect is particularly pronounced for type A agents, while type B agents show only a small decrease in performance with increasing initial distance to the target. In any case, it is worth noting that the policy learned by constraining the initial distance to the annular region defined by *σ*/2 < *r*_ini_ ≤ *R̃* remains valid even when the initial distance exceeds *R̃*. While this is expected for type B and C agents, which perform temporal comparisons also for *r* > *R̃*, the result is less obvious for type A agents. In the latter case, it is the consequence of a higher tumbling probability learned in the external region, see discussion below.

**Fig. 3:**
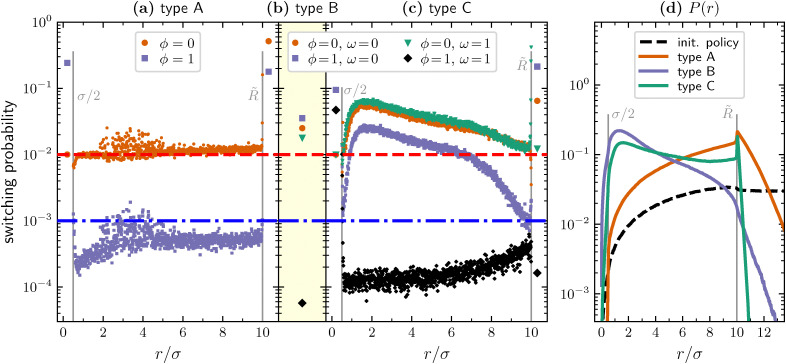
(a) Switching probabilities from passive to active phase (orange circles) and from active to passive phase (blue squares) for type A agents at episode 10^6^. (b) For type B agents at episode 10^6^, switching probabilities from passive to active phase with *ω* = 0 (orange circle) and with *ω* = 1 (green triangle), and from active to passive phase with *ω* = 0 (blue square) and with *ω* = 1 (black diamond). (c) Same as in panel (b) but for type C agents. In panels (a)–(c), we also report the initial probability of switching from the passive to the active phase (dashed red line) and from the active to the passive phase (dash-dotted blue line). In panels (a) and (c), the value of the switching probability inside the target (*r* ≤ *σ*/2) and for *r* > *R̃* are reported with a larger symbol and the grey vertical lines help distinguish the three regions: *r* ≤ *σ*/2, *σ*/2 < *r* ≤ *R̃*, and *r* > *R̃*. (d) Radial distribution of the position of the different agents during target search events. Statistics obtained over 10^5^ target-search events adopting the policies reported in panels (a)–(c). Normalization is such that 
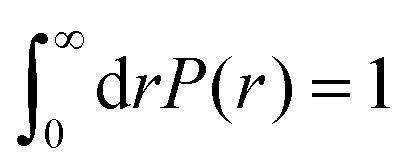
.

To gain a deeper understanding of the behavior of the trained agents, we can leverage the interpretability nature of the PS scheme and directly examine the learned policy, *i.e.* the probabilities of switching phase given their state. These are computed starting from the matrix encoding the learning process (the *H*-matrix, see Methods section for details), averaged over different agents, and are reported in Fig. 3a, b, and c for type A, type B, and type C agents, respectively.

Starting with type A agents (Fig. 3a), we can observe that the switching probability inside the target area is very high when the agent is in the active phase (*ϕ* = 1) and is equal to the relative value of the initial policy when the agent is in the passive phase (*ϕ* = 0). Intuitively, these observations are respectively in line with the facts that the agent cannot detect the target while being in the active phase, thus willing to become passive as soon as it enters the target area, and that when the agent finds the target the episode ends and the *H*-matrix value corresponding to *ϕ* = 0 and *r* ≤ *σ*/2 is thus never updated. We also note that the switching probability is very high in both phases when *r* > *R̃*, meaning that, when its distance from the target becomes too large, the agent tumbles frequently, attempting to find a favorable run direction that brings it back to the region where it is able to better resolve the distance from the target. In the region defined by *σ*/2 < *r* ≤ *R̃*, we can at first notice that the switching probability when being in the passive phase remains close to its initial value while the switching probability when being in the active phase is generally smaller than its corresponding initial one, meaning that active phases have to be on average longer than what initially defined. Furthermore, we see a quite large volatility in the values of both the switching probabilities, especially for intermediate distances between 2*σ* and 5*σ*. This observation is likely due to the fact that different agents learn quite different policies in terms of the specific value of the switching probability corresponding to the various distance bins and that this value for 2*σ* ≲ *r* ≲ 5*σ* is affecting relatively less the agent performances. A more careful inspection shows a small drop of the switching probability for *ϕ* = 0 when approaching *r* = *σ*/2 and a corresponding increase of the one for *ϕ* = 1. This results in the agents willing to have a relatively more passive behavior (which allows target detection) in the close vicinity of the target. Finally, we notice a small increase in both the probabilities, resulting in relatively more frequent tumbles, just before reaching the distance *r* = *R̃*.

Type B agents cannot discriminate their distance to the target. Thus for each *ϕ* and *ω* there is only one value of the switching probability and the resulting policy, reported in Fig. 3b, is readily interpretable. In particular, regardless of *ω*, the passive-to-active switching probability is larger than its initial value. On the other hand, the active-to-passive probability is high when the particle moves away from the target (*ω* = 0) and low otherwise (*ω* = 1). This ensures that active phases driving the agent in a favorable direction are prolonged, whereas tumbling becomes more frequent when the agent moves unfavorably.

When considering the policy learned by type C agents (Fig. 3c) we can detect similar features to those previously detailed for type A agents. Namely, a large probability to switch from active to passive inside the target area, increased frequency of tumbling events in the outer region, and a relatively large spread of the probabilities values in between these two extrema. However, the introduction of the additional perceptor comes with new features not displayed by the first kind of agents. Firstly, although there is no immediately obvious distinction between the probability of switching from the BP to the ABP phase when the particle is getting closer to the target (*ω* = 1) or not (*ω* = 0), type C agents further reduce the duration of the passive phase. This enables them to spend more time in the active phase, where they can take advantage of the additional information provided by the extra perceptor. Indeed, the probability of switching from the ABP to the BP phase is clearly distinct if *ω* = 0 or *ω* = 1. Agents that are active and are getting closer to the target decrease their switching probability, such that they can exploit their self-propulsion to decrease even more their distance from the target. In contrast, agents that are active but are not getting closer to the target increase the switching probability with the aim of tumbling and starting as soon as possible a new run eventually having a more favorable direction.

Finally, we note that the probability of switching from the BP to the ABP phase, just before *r* = *R̃*, shows a drop if *ω* = 0 and an increase otherwise. This behavior is not easy to interpret because one would expect that, being each step in the passive phase in a random direction, there should be no difference in the switching probabilities shown for *ω* = 0 and for *ω* = 1. However, the variable *ω* is evaluated as a temporal comparison between two different time integration steps and there is a certain probability that the phase at the two times differs. Furthermore, in PS the rewards are propagated back in time through the glow matrix (see Methods section for details). Thus, favorable or unfavorable actions taken at a certain time, affect the update of the *H*-matrix not only for the current state-action pair but also for those met later. Thus, there is a non trivial interplay between the switching probability observed for the varius states that leads to the observed behavior and that we are unable to unravel completely. Stated differently, this particular feature of the learned policy is complex enough to evade the common notion that, unlike supervised and unsupervised machine learning, RL algorithms allow interpretability of the learned strategies.

All these considerations find their counterpart in the probability of being at a certain distance from the target during an episode, see Fig. 3d. Since at the beginning of an episode the agents are introduced uniformly within the region defined by *σ*/2 < *r* ≤ *R̃* and the episodes have a limited duration, the radial probability obtained by following the initial policy increases with *r* in such a region, and has a very slow decay for *r* > *R̃*. On the other hand, the same observable computed for optimized type A agents shows a similar behavior for *r* ≤ *R̃* but a quick drop in the external region, suggesting that most of the success achieved by these agents is due to their ability to perform quick tumbles once they are beyond the detection range and prolonged runs in the region where they can detect the distance from the target. Remarkably, the radial probability displayed by type B and type C agents not only has an even faster decay for *r* > *R̃* but it starts to decrease already for *r* ≳ 2*σ*. Finally, note that for both type A and type C agents the increased tumbling rate in the external region results in a peak of the radial distribution just beyond the detection range.

**Fig. 4:**
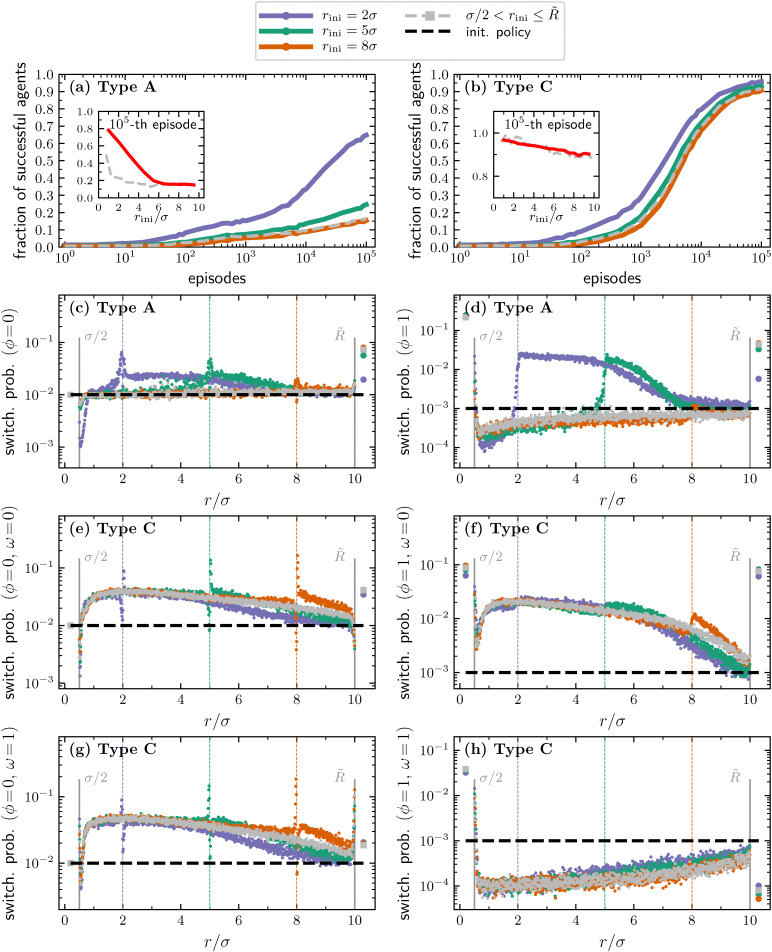
(a) Fraction of successful type A agents as a function of the number of episodes, shown for various fixed initial distances from the target. The learning curve for *r*_ini_ uniformly randomized within the range *σ*/2 < *r* ≤ *R̃*, previously shown in Fig. 2a, is here again reported (grey-dashed line). Inset: Fraction of successful agents as a function of the initial distance. (b) Same as panel (a) but for type C agents. (c)–(h) Switching probabilities at episode 10^5^ for *r*_ini_ = 2*σ* (violet), *r*_ini_ = 5*σ* (green), and *r*_ini_ = 8*σ* (orange). Across panels (c)–(h), the corresponding initial policy is reported with a black-dashed horizontal line, the value of *r*_ini_ is marked with a vertical dashed line of the corresponding color, the policy learned leaving *r*_ini_ uncostrained is reported with gray squares, and the grey vertical lines help distinguish the three regions: *r* ≤ *σ*/2, *σ*/2 < *r* ≤ *R̃*, and *r* > *R̃*.

The results reported above show that, for type A and C agents, the detection range *R̃* plays an important role in learning a successful strategy, whereas the fact that the switching probabilities take similar values in both regions *σ*/2 < *r* ≤ *R̃* and *r* > *R̃* suggests that it plays a minor role for type B agents. An interesting question is whether our learning agents are able to adapt and benefit from a second imposed length scale, as suggested by previous literature.^54–56^ We explore such a question by running our learning algorithm for type A and C agents in a setup in which the distance from the target at the beginning of each episode, *r*_ini_, is fixed. Specifically, we run 18 different learning processes at fixed *r*_ini_, varying its value in the range from *σ* to 9.5*σ* in steps of 0.5*σ*. Fig. 4 show the results obtained for three of the different values of the initial distance, namely *r*_ini_ = 2, 5,  and  8*σ*, after a learning process lasting 10^5^ episodes. In line with what already observed in ref. 54, we first note that the learning performances displayed by the learning agents improve as the initial distance decreases, see Fig. 4a and b. This effect is more pronounced for type A agents which are unable to make temporal comparisons. After 10^5^ episodes, type C agents with a fixed *r*_ini_ reach a fraction of successful agents as a function of the initial distance which is similar to that obtained with the original protocol having *r*_ini_ randomly chosen in (*σ*/2,*R̃*], see inset of Fig. 4b. This does not hold for type A agents, where the protocol with a fixed *r*_ini_ yields improved results at the 10^5^ episode, provided that *r*_ini_ ≲ 6*σ*, see inset of Fig. 4a.

The policies learned after 10^5^ episodes clearly show the fingerprint of the newly imposed length scale (Fig. 4c–h). Indeed, both the probabilities of switching from passive to active and from active to passive for type A agents peak in correspondence of *r*_ini_ and have increased values (with respect to the initial policy) for *r* > *r*_ini_. This indicates that type A agents tend to tumble more frequently as they move beyond their initial distance from the target. In particular, the large value of the probability of switching from active to passive for the states with *r* > *r*_ini_ and its slow decrease towards the initial policy (see Fig. 4d) point to the fact that the agents aim at shortening the active phases that are bringing them further from the target. The signature of the imposed initial distance is less pronounced in the case of type C agents. Specifically, the probability of switching from active to passive while getting closer to the target (*ω* = 1) is equivalent to that learned in the protocol in which the initial distance is not fixed (Fig. 4h). With the exception of the case *r*_ini_ = 8*σ*, which is probably due to the different learning rates among the various cases, the same holds also when the state of the agents is such that, in the last integration step, it has moved further from the target (*ω* = 0), see Fig. 4f.

Regarding the learned probability for switching from the BP to the ABP phase, we note that, if *ω* = 0 it is quite similar to the one learned when the initial distance is not fixed but it shows a drop just before *r* = *r*_ini_ and an increase just after (Fig. 4e). The opposite holds for *ω* = 1 (Fig. 4g). This behavior is similar to what is already observed in the proximity of *R̃* for the policy learned in the case in which the initial distance is not fixed. Again, we failed to find a complete explanation for this observed behavior but we note that the agents are able to reproduce it also at the new imposed length scale, corroborating the belief that this behavior plays an important role in optimizing the performances of type C agents.

## 4 Conclusion

When looking for nourishment, bacteria like *E. coli* make no controlled changes of direction. However, they can sense the concentration of a certain chemoattractant, compare its value to a previous one, and adjust their tumbling rates accordingly. This results in a run-and-tumble motion characterized by longer runs in the favorable direction, thus enhancing their efficiency in reaching the target.

We tried to better understand how this bacteria behavior arises by exploiting reinforcement learning. In particular, we considered a learning agent initially performing unbiased run-and-tumble dynamics and applied the projective simulation algorithm^44^ to enable the agent to develop an efficient stochastic target-search strategy. The learning agent has been modelled as an intermittent active Brownian particle^26,43^ that can switch from a standard passive Brownian phase to an active Brownian phase and *vice versa*. The transition between these phases occurs with a probability that depends on the agent's internal state and that is tuned during the learning process in order to optimize the target-search performances. We considered three different types of agents: type A agents can only sense the distance to the target, which in a homogeneous environment is a good proxy for the concentration of ligands released by the target. Type B agents, cannot directly exploit the absolute value of the distance to the target, but are endowed with a short time memory that allows them to make temporal comparisons similar to those made by bacteria. Finally, type C agents can both exploit the absolute value of the distance to the target and perform relative comparisons of it on a short time scale.

Our findings show that all types of agents are able to learn successful target-search policies, with those equipped with temporal comparison abilities achieving significantly better performances. This is partly expected when the relocation phase is fast and suggests that the ability to perform temporal comparisons of ligand concentrations is more important than the ability to evaluate their absolute values, and it may explain why evolution has equipped bacteria with the former. Furthermore, contrary to what is displayed by type A agents, the efficiency of trained type B and C agents only slightly depends on the initial distance of the target. By inspecting the policy learned by type A agents we note that the probability of tumbling increases with the distance to the target and that the active phases should in general be longer than what initially defined based on the *E. coli* behavior in a uniform dilute acqueus medium.^2^ The additional information sensed short-time comparison made by type B and C agents results in a clear distinction in the switching probability from the active to the passive phase: depending on whether the particle is moving towards the target or not, the active phases are respectively prolonged or shortened. Finally, when the initial distance of the target is fixed during the learning process, the policies learned by type A and C agents clearly display a signature of this additionally imposed length scale. Consequently, this agents learn to exploit this additional length scale to further improve their efficiency in locating the target, in accordance with what is suggested by previous literature.^54–56^

Our work is mainly addressed to investigating how the chemotactic behavior shown by bacteria can be achieved through reinforcement learning. However, the same framework can also be applied to artificial microswimmers whose activity can be controlled by an external illuminating system.^38^ While it has already been shown that Janus particles^52^ able to couple their self-propulsion orientation to a chemical gradient can perform chemotaxis,^57^ the phase switching mechanism we proposed may represent a valid alternative to endow artificial microswimmers with chemotactic abilities. This further justifies our investigation of type A agents, as it may be simpler to endow synthetic or robotic systems with the ability to measure the distance to a target rather than to perform temporal comparisons.

Finally, our work can be leveraged to explore more complex and eventually realistic scenarios such as, for instance, bacterial migration through confined spaces and porous media^58–60^ or in front of solid surfaces.^61,62^ Moreover, the randomization of the self-propulsion direction at each phase switch could be suppressed in order to take into account that there are experimental indications that bacteria can also tune the amount of reorientation during the tumbling phases by controlling their duration. Moreover, the randomization of the self-propulsion direction at each phase switch could be suppressed to reflect experimental evidence suggesting that bacteria can modulate the amount of reorientation during tumbling by adjusting the duration of this phase.^50,51^

## 5 Methods

To identify effective target-search strategies, we employed the reinforcement learning algorithm projective simulation (PS). Originally developed for designing autonomous quantum learning agents,^44^ PS has demonstrated competitive performance in classical RL problems as well^63,64^ and has been successfully applied to other target-search-related problems.^26,54,65^

The key feature of the PS algorithm is the use of a particular memory structure, termed episodic and compositional memory (ECM), mathematically represented as a graph of interconnected units called clips. Clips correspond to either perceptual units (state), decision units (actions), or a combination of those.

We modeled the target-search problem as a Markov decision process,^21^ where, at each learning step, the agent has a state *s*, performs an action *a* based on a policy defined by the conditional probabilities π(*a*|*s*), and receives a reward ℛ as feedback. The ECM structure in this context consists of a layer of states fully connected to a layer of actions. Each state-action pair (*s*,*a*) is associated with a real-valued weight *h*(*s*,*a*), called the *h*-value, which defines the policy as:4
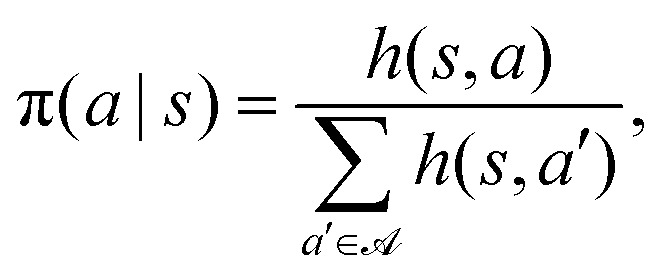
where 𝒜 is the set of all possible actions. Additionally, a non-negative glow value *g*(*s*,*a*) tracks the frequency and recency of visits to specific state-action pairs, influencing the policy updates to optimize the total expected reward. This glow-based updating mechanism makes PS particularly well-suited for our target-search problem: since a large number of iterations of the motion eqn (1)–(3) are required before finding the target and receiving a reward, the reward signal is sparse and weakly correlated with individual state-action pairs. Methods capable of processing sequences of state-action pairs, such as PS, are thus more effective than traditional action-value algorithms like Q-learning or SARSA,^21^ which failed to produce successful policies in a similar setup.^26^

In our model (see Dedicated section), the action *a* is binary: *a* = 1 triggers a phase switch, while *a* = 0 maintains the current phase. Applying the PS framework with a learning iteration at each integration of the equations of motion, eqn (1)–(3), each learning step proceeds as follows:

• The agent determines the phase-switching probability *p*_*t*_ for the current state *s*_*t*_ as:5
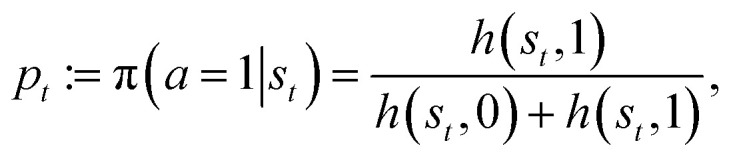
and selects the next phase *ϕ*_*t*+Δ*t*_ accordingly;

• The glow matrix *G* is damped according to

6 *G* ← (1 − *η*)*G*,

where *η* is called the glow parameter and determines how much a delayed reward should be discounted;

• A unit is added to the glow value of the current state-action pair

7 *g*(*s*_*t*_,*a*_*t*_) ← *g*(*s*_*t*_,*a*_*t*_) + 1;

• The particle's position and sef-propulsion direction at time *t* + Δ*t* are computed according to eqn (2) and (3);

• The *h*-value matrix *H* is updated as

8 *H* ← (1 − *γ*)*H* + *γH*_0_ + ℛ*G*,

where ℛ is the reward (being 1 if *r*_*t*+Δ*t*_ ≤ *σ*/2 and 0 otherwise), and the damping parameter *γ* controls the rate of return to an initial matrix *H*_0_.

The glow and damping parameters are treated as hyperparameters and tuned for optimal learning performance. Along the manuscript we used (*γ*,*η*) = (10^−6^, 10^−4^) both for type A and type C agents.

To ensure a finite number of states, the distance to the target *r* is binned using a bin width equal to 0.01*σ* in the interval *σ*/2 < *r* ≤ *R̃* and two more bins are defined for *r* ≤ *σ*/2 and *r* > *R̃* respectively.

The initial policy assigns probabilities of phase switching as 10^−2^ and 10^−3^ for passive and active phases, respectively, achieved by setting the *h*_0_-values accordingly.

The time step is Δ*t* = 10^−4^*τ*.

Similarly to what has been done in a previous paper,^26^ we optimize computational efficiency by exploiting the fact that the reward is different from zero only when the target is found and that finding the target terminates the current episode. We then update the *H*-matrix only at the end of an episode according to9
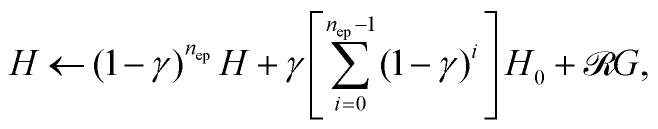
where *n*_ep_ is the number of learning steps within the given episode. During intermediate steps within the episode *n* := *t*/Δ*t* ≤ *n*_ep_, the phase-switching probability *p*_*t*_ is computed according to10
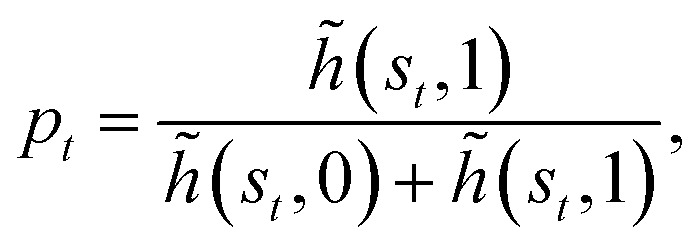
using the temporarily updated *h*-values:11
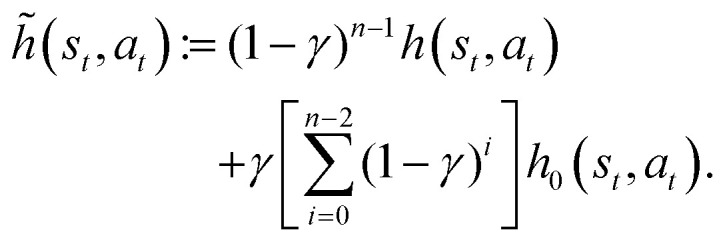


It is also computationally extremely more efficient to save, for each state-action pair (*s*,*a*), the time steps 
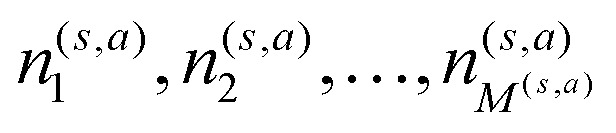
 at which they are visited and then also update the glow matrix only at the end of the episode according to12
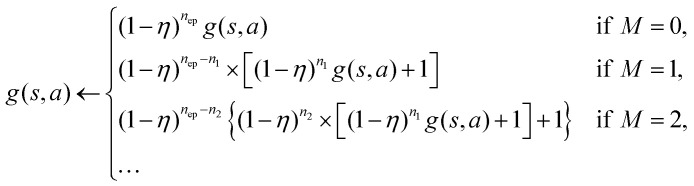
where we dropped the superscripts (*s*,*a*) for the sake of compactness. All elements of the *G* matrix are initialized to zero every 20 episodes.

## Author contributions

N. T. and M. C. developed the software and analyzed the results. All authors conceived the research and wrote and reviewed the manuscript.

## Conflicts of interest

There are no conflicts to declare.

## Data Availability

The software and scripts necessary to reproduce data contained in this manuscript can be found at https://researchdata.uibk.ac.at/records/0zgr8-j9426.
